# Recent Progress in Surgery

**Published:** 1877-07

**Authors:** 


Recent Progress in Surgery. — *Excision of Joints.*—Dr. Culbertson has collected his cases by means of circulars addressed to physicians in the United States and reference to various surgical treatises and medical journals. The work is chiefly statistical in its character.

*Excision of the Hip-Joint.*—One hundred and twenty-one cases of excision for gunshot injury have been collected. Of one hundred and nineteen of these, of which the result is known, thirteen recovered. The results given show that this excision cannot be strongly advocated with a view of obtaining useful limbs, but simply as a surgical resource to save life.

Four hundred and seventy-two cases of excision for disease are given. Of these two hundred and forty-one cases of partial excision. In regard to the extent of bone removed, we learn that the greatest mortality was shown in cases in which the head of the femur alone was excised, next the head and neck, next head, neck, and trochanter. The most favorable results were obtained when the head and part of the trochanter were removed. There are one hundred and seventy-seven cases of complete excision for disease. We find here that the mortality is greater in complete than in partial excisions; that it increases as the amount of pelvic bone is removed. "One might say that the mortality-center is at the head of the femur, the rate diminishing as the bone is removed outwards along the femur, but increasing as it advances inwards upon the pelvis." Ninety cases of this series recovered. Forty-five per cent. of these obtained "perfect limbs."

*Excision of the Knee-Joint.*—Mr. Filkin, of Northwich, England, was the first to execute a complete excision of this joint for disease, 1762. The author has collected about seven hundred cases. In comparing hospital with private practice we find that 25 per cent. died in private practice and 87.5 per cent. in hospital practice, when the operation was done for gunshot injury. When done for disease the mortality in private practice was 30.76 per cent. and in hospital practice 25 per cent. Males endure this operation better than females, according to these statistics. For gunshot wounds the most favorable period of life for the operation is from fifteen to twenty years. In disease the most favorable is from five to fifteen years. The form of incision made no essential difference in the mortality. In gunshot injuries 58.82 per cent. gained useful limbs, and in 23.52 per cent. the limbs were worthless. In excision for disease, four hundred and twenty cases recovering and eleven dying, 14.38 per cent. had "perfect" and 42.45 per cent. useful limbs.

*Excision of the Ankle-Joint.*—Two hundred and eighty-five cases for disease, injury, or deformity are reported, and forty-five for gunshot injury. The mortality is greater in gunshot wounds than in injuries; there is no mortality among the cases of excision for disease and deformity in the "traumatic," non-traumatic, malignant, or syphilitic cases. The author says that "it is evident a large proportion of these excisions result in more or less usefulness of the members."

*Excision of the Shoulder-Joint.*—About a thousand cases are included under this head. A little over 2 per cent. gained perfect limbs in gunshot injuries, and 22 per cent. had useful members. In 1.03 per cent. the extremities were worthless. In the class of injuries 13 per cent. secured perfect results. In diseases perfect results were obtained 9.47 per cent., and 70.52 per cent. were useful, 4.15 per cent. were worthless. A

few cases of subperiosteal excision are given, and we find in one case where 4.71 inches of the bone was removed there was but 1.17 of an inch shortening. In a second case where 3.93 inches of bone was excised, there was .75 of an inch shortening only. It is to be regretted that the question of subperiosteal resection is not considered in a special table for all the joints, but we presume the number of these cases were too few to admit of a fair comparison.

*Excision of the Elbow-Joint.*—The most favorable age for excision of this joint for disease was found to be from ten to twenty years. But about five per cent. of the patients died of this operation. A large per cent. gained useful limbs. The results as to usefulness are more satisfactory in the partial than in the complete excision.

*Excision of the Wrist-Joint.*—The single longitudinal incision gave the most favorable results, and the double lateral incision and Lister's modification of the same gained useful members next in order. In disease perfect results were obtained in 7.50 per cent. of the cases, 45.57 per cent. gained useful limbs, and in 24.02 per cent. the members were worthless.

The operation known as Lister's is described in detail, and it will be remembered that in a former report this operation was mentioned as highly praised by Dr. Otis.

In a report of the sixth congress of the Society of German Surgeons is a discussion on resection of joints, brought out by a paper from Professor Huter. His general conclusion as regards resection of the foot was that partial resection must be generally practiced in military surgery, but that great caution was necessary in employing it in caries. In regard to the elbow-joint he sums up as follows: (1.) In cases of injury, partial resection is almost always to be performed. (2.) In caries great caution is necessary in undertaking resection, and a decision as to its results in respect to function has yet to be arrived at. Professor Gurlt believed that anchylosis was more frequent in partial resection of this joint. Langenbeck thought that in cases of injury one could not be too conservative; on the other hand, in disease it was very important to remove every portion of diseased bone. A discussion arose as to the propriety of excising or leaving the trochanter in resection of the hip-joint. Mr. Volkmann preferred removing the trochanter, there being liability of anchylosis if the head alone was excised. Dr. Lucke, of Strasburg, made the removal of the trochanter major dependent only on the adequate escape of the secretions of the wound. In young individuals he only partially removed it. Dr. Langenbeck disapproved of the principle of removing the trochanter. Most of the resections were performed on children, and the growth of the bone was interfered with by dividing it below the trochanter. He was not much in favor of long drainage tubes, but they were often necessary in this operation, and he had even introduced them through the acetabulum into the pelvis. Dr. Volkmann also used long drainage tubes. He was of the opinion that the simple removal of the head of the femur had as much influence on the growth of the bone as the division below the trochanter; the femur grew from the lower end. In his cases growth was not arrested,, and a useful new joint was more easily obtained by his method, as the sawn surface of the femur, after the excision of the trochanter, came into contact with the acetabulum as if it were a new head of the bone.

In the Boston City Hospital Report, just published, is an interesting account, by Dr. D. W. Cheever, of a dissection of a newly formed elbow-joint seven years after a subperiosteal excision performed by him. The patient was a boy fourteen years of age at the time of the operation. From the sawn ends of the shafts of the bone had developed: "(1.) A new head of the radius. (2.) A new coronoid process firmly attached, a new (rudimentary) olecranon partially attached to the ulna. (3.) A long, powerful hook of bone representing the external condyle of the humerus, and firmly attached to the shaft. (4.) A large, partially detached and irregular bone representing the internal condyle and trochlea. (5.) The articular end of the humerus, trochlea, and lesser head, imperfectly repaired, but in process of bony growth." All the bone sections in the operation were made in the shafts above the line of the epiphyses. The arm was a very useful one. Two photographs give an excellent idea of the extent of reproduction of bone.

*"New" Operation for Openiug the Nasal Cavity.*—Mr. Harrison Cripps devised the method for removing the sequestra of bone from the nose of a syphilitic female aged thirty. The fœtid discharge had ulcerated the skin of the lip and made her an object of aversion. The operation is thus described: "The right corner of the upper lip was seized by the operator and the left by his assistant; by this means the lip was everted and drawn upwards, while the soft parts were separated by a clean sweep of the scalpel, cutting upwards with its edge kept close to the bone. This incision extended from the second bicuspid tooth on the right side to that on the left. By drawing upon the upper lip, the nose, together with the soft parts forming the anterior portion of the face, could be easily raised in such a manner as thoroughly to expose the nasal fossæ. . . . After the removal of the bone, the forefinger could be passed quite back to the posterior wall of the pharynx. . . . There was scarcely any bleeding during the operation." This operation has been described by Rouge, who has performed several; also by Mr. Warrington Howard, Messrs. Pollock and Holmes.

*Rapid Cure of Popliteal Aneurism by Esmarch's Bandage.*—Dr. Thomas Smith, F. R. C. S., reports the case. The patient was a man aged forty-five. The swelling was about the size of a hen's egg, and was of three weeks' duration. "It came of itself." It was increasing pretty rapidly. Pressure on the femoral controlled the pulsations readily, but flexions of the limb did not affect them. There was severe pain at night. After a few days' rest in bed, on March 17th, at three P.M., "the limb was rolled in a flannel bandage from the toes to the lower part of the popliteal space, and again from above the aneurism to the groin. Esmarch's india-rubber bandage was then applied, with only moderate firmness, from the toes to the aneurism, the patient being in bed; he was then made to stand up until the sac was well filled with blood, when the elastic bandage was applied from above the aneurism to the groin, where the limb was surrounded with the thick india-rubber tubing, so as completely to arrest the circulation in the limb. The aneurism and popliteal space were thus left exposed, so that the least pulsation in the sac could be detected. In this way the circulation was stopped for one hour, during the last half of which chloroform was used on account of pain. At the end of the hour, while the patient was still under chloroform, Esmarch's bandage was removed, and the Italian tourniquet was applied to the femoral and maintained in position for two hours." It was finally removed to relieve pain, and the aneurism was found to be reduced in size, and solid. He was discharged April 6th, cured.

A number of cases are reported in this article, most of which were successful; a brief summary of them is given. The indications are to arrest the circulation in the limb, and, at the same time, keep the sac filled with blood. Each operator has used slight modifications of the above. In a previous report we have already quoted some of these cases. We presume that aneurisms of any considerable size would hardly be amenable to this treatment. We should expect more frequently in the latter case a satisfactory result from compression by the Massachusetts General Hospital tourniquet.—*Boston Medical and Surgical Journal.*

